# Editorial: Genetic research into neurodegenerative disorders

**DOI:** 10.3389/fneur.2024.1367627

**Published:** 2024-01-29

**Authors:** Xiaoting Zheng, Jianhai Chen, Chunyu Li

**Affiliations:** ^1^Department of Neurology, Laboratory of Neurodegenerative Disorders, National Clinical Research Center for Geriatrics, West China Hospital, Sichuan University, Chengdu, Sichuan, China; ^2^Laboratory of Neurodegenerative Disorders, National Clinical Research Center for Geriatrics, West China Hospital, Sichuan University, Chengdu, Sichuan, China

**Keywords:** genetics, Parkinson's disease, Alzheimer's disease, hereditary spastic paraplegia, rare variant

The integration of advanced sequencing technologies into genetic research has ushered in a new era of understanding the intricate genetic foundations of neurodegenerative disorders. This transformative approach has yielded a wealth of insights, emphasizing the pivotal role played by genetic factors in susceptibility to conditions such as Parkinson's disease (PD), hereditary spastic paraplegia, and Alzheimer's disease (AD).

In a study on PD, Liu et al. utilized burden genetic analysis combined with high-throughput sequencing to evaluate Dynamin-1-like (*DNM1L*) variants in the Chinese population. They identified 23 rare variants and 201 common variants in the *DNM1L* coding region but found no significant relationship with PD susceptibility, thereby broadening the genetic spectrum of the *DNM1L* gene in PD. The results not only expanded our knowledge of the genetic diversity within the *DNM1L* gene, but also significantly advanced our understanding of its implications for PD susceptibility.

Similarly, Ferese et al.'s investigation into hereditary spastic paraplegia type 4 (SPG4) patients with *SPAST* gene mutations has employed next-generation sequencing panels to uncover a rich tapestry of splice variants. The combined approach of *in silico* and *in vitro* analysis for splicing mutations confirmed the pathogenic mechanism of two splicing variants (i.e., c.1245+1G>A and c.1414-2A>T).

In the context of AD, Wang et al.'s innovative use of optimized transcriptome-wide association studies has not only expanded our knowledge base with the identification of 415 associated risk genes but has also led to the validation of 34 highly reliable biomarkers, including *APOC1, CR1, ERBB2*, and *RIN3*. This not only corroborates existing knowledge but also illuminates novel avenues for deeper exploration into AD pathogenesis.

Furthermore, Cox et al. described the clinical spectrum of mitochondrial encephalomyopathy, lactic acidosis, and stroke-like episodes (MELAS), encompassing both patients and asymptomatic carriers. They observed varying heterogeneity levels in different tissues and found distinct manifestations in standard and late-onset cases with similar or divergent genetic mutations.

Collectively, these multifaceted investigations underscore the intricate interplay of genetic factors in neurodegenerative disorders, urging the scientific community to embark on further explorations to uncover novel risk variants, elucidate underlying mechanisms, and delineate intricate pathways. Such analyses are essential for advancing our knowledge and developing targeted interventions for these complex and challenging conditions.

## Author contributions

XZ: Writing—original draft. JC: Writing—review & editing. CL: Writing—review & editing.

